# A Novel Aspect of Essential Oils: Coating Seeds with Thyme Essential Oil induces Drought Resistance in Wheat

**DOI:** 10.3390/plants8100371

**Published:** 2019-09-25

**Authors:** Maissa Ben-Jabeur, Rubén Vicente, Camilo López-Cristoffanini, Noura Alesami, Naceur Djébali, Adrian Gracia-Romero, Maria Dolores Serret, Marta López-Carbonell, Jose Luis Araus, Walid Hamada

**Affiliations:** 1 Laboratory of Genetics and Plant Breeding, National Institute of Agronomy of Tunis, 43, Av Charles Nicolle, Tunis 1082, Tunisia; 2 Max Planck Institute of Molecular Plant Physiology, 14476 Potsdam-Golm, Germany; vicenteperez.ruben@gmail.com; 3 Departament de Biologia Evolutiva, Ecologia i Ciències Ambientals, Secció de Fisiologia Vegetal, Universitat de Barcelona, 08028 Barcelona, Spain; camilo.lopez.cr.puc@gmail.com (C.L.-C.); mlopez@ub.edu (M.L.-C.); 4 Faculty of Agriculture, Damascus University, Damascus 306, Syria; nouraalesami1992@gmail.com; 5 Centre of Biotechnology of Borj Cedria, Laboratory of Bioactive Substances, BP 901, Hammam-Lif 2050, Tunisia; 6 Section of Plant Physiology, University of Barcelona, 08028 Barcelona, Spain; adriangraciaromero@hotmail.com (A.G.-R.); dserret@ub.edu (M.D.S.); 7 AGROTECNIO (Center of Research in Agrotechnology), University of Lleida, 25198 Lleida, Spain

**Keywords:** thyme essential oil, coating, drought, wheat, resistance, isotope

## Abstract

Coating seeds with biostimulants is among the promising approaches in crop production to increase crop tolerance to drought stress. In this study, we evaluated the potential of coating durum wheat seeds of the cultivar ‘Karim’ with thyme essential oil on enhancing seed germination and seedling growth, and on plant growth promotion and induction of drought resistance. Coated seeds were pre-germinated, grown in hydroponics, and grown in pots under controlled well-watered and progressive water/nutrient stress conditions. Seed coating with thyme oil increased germination rate and enhanced seedling growth development in hydroponics. In the pot experiment, thyme oil increased, when well watered, root and shoot development, chlorophyll, nitrogen balance index (NBI), abscisic acid (ABA), anthocyanins and flavonoids in leaves, decreased nitrogen isotope composition (δ^15^N) and increased carbon isotope composition (δ^13^C) of shoots. Increasing water/nutrient stress in control plants induced higher accumulation of ABA and anthocyanins coupled with a transient decrease in chlorophyll and NBI, a decrease in shoot and root development, the Normalized Difference Vegetation Index (NDVI), shoot C content, δ^15^N, and an increase in δ^13^C, revealing the avoidance strategy adopted by the cultivar. Thyme oil had the potential to enhance the avoidance strategy by inducing roots elongation, reducing the loss of shoot and roots dry matter and chlorophyll, maintaining balanced NBI, an decreasing anthocyanins, flavonoids, and δ^13^C via maintaining lower ABA-mediated-stomatal closure. Thyme oil increased shoot N content and δ^15^N indicating preferential uptake of the ^15^N enriched NH_4_^+^. Coating seeds with thyme oil is suggested as a promising alternative approach to improve plant’s water and nutrient status and to enhance drought resistance.

## 1. Introduction

Globally, wheat is considered the most important grain source for humans and particularly the most cultivated crop in the Mediterranean region [1]. One of the major constraints affecting wheat production in the Mediterranean region is drought [2]. Water stress induces a decrease in leaf water potential, stomatal closure resulting in reduced CO_2_ assimilation, increased production of ROS, damage of photosynthetic pigments, reduced photosynthesis, loss of membrane integrity, cellular death, and, if severe, causes a significant decrease in plant productivity. Facing a water deficit situation, plants apply an array of morphological, physiological, and biochemical adaptations in order to prevent oxidative stress and avoid the related cellular damage: (i) Production of osmo-protective and antioxidant secondary metabolites, able to scavenge and detoxify ROS such as flavonoids and antioxidant enzymes; (ii) hormonal accumulation, mainly of abscisic acid (ABA) which triggers a cascade of physiological responses, including ROS production, increase in cytosolic Ca^2+^, protein phosphorylation, dephosphorylation events, and stomatal control over transpiration; and (iii) morphological changes, associated to hormone actions, involving an increase of the root to shoot ratio and of parenchyma thickness, and a decrease of leaf number and leaf area to minimize transpiration rates [3,4].

Taking into account the ongoing rise of climate change, the increasing food demand for rising population and the high consumption of wheat-based food, research has been focused on multiple approaches to overcome crop failure [5]. These approaches include breeding and agronomical tools to improve crop resilience to drought, and at a different scale the design of integrated water policies and technologies for advanced water use efficiency [2]. Some of these approaches are not economically viable, either expensive or time consuming, which sustain rapid achievement of crop adaptation to drought. Seed priming strategy through the technique of seed coating with natural biostimulant compounds is a promising approach which may enhance germination, establishment of seedlings, nutrient uptake [6,7], and as a result, plant tolerance to biotic and abiotic stresses [8]. Better crop establishment and resource use efficiency tends to save time and outweigh expenses for farmers, including the need of monitoring crop performance during the whole growing cycle and the uncertainty of the effectiveness of some approaches, [9] like a broad distribution of fertilizers. On the basis of our previous reported work on the potential of thyme essential oil to prime seedlings when applied at roots [10], the effect of coating wheat seeds with thyme oil on seed germination, seedling emergence, and plant growth promotion and adaptation to water/nutrient stress is the subject of the current research. The number of studies focusing on drought stress during early growth is limited, despite the occurrence of drought during this period [11]. Furthermore, plant responses to water/nutrient stress and biostimulant effectiveness are most likely modulated by stress severity. Therefore, different water/nutrient regimes were applied in this study to wheat plants at the vegetative growth stage, projecting early-season drought severities, with the aim to forecast biostimulant effects on plant’s adaptive response, when subjected to different levels of drought stress in field conditions.

## 2. Results

### 2.1. The Effects of Seed Coating with Thyme Oil on Seed Germination and the Root and Shoot Development of Wheat Seedlings

**Figure 1:**
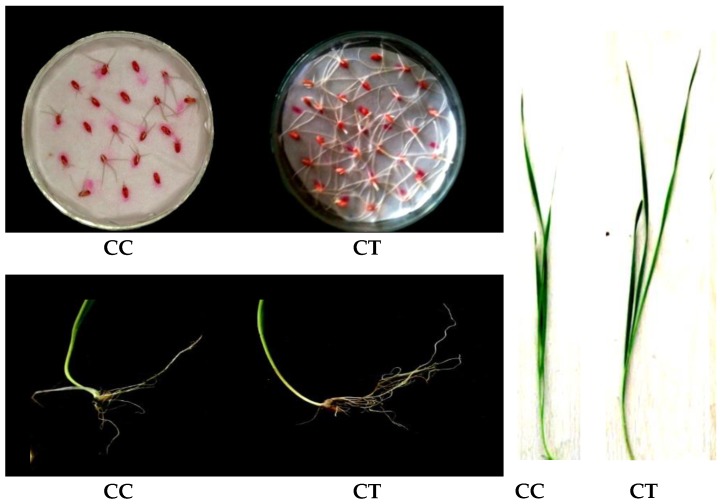
The Effects of thyme oil on seed germination and root and shoot early growth of wheat plants. CC: Coated control; CT: Coated with thyme oil.

The analysis of variance (Table 1) showed that seed coating significantly affected seed germination and the root and shoot development (*p* < 0.001), in wheat seedlings. The results showed that seed coating with thyme oil significantly increased the germination rate (100%) compared to the control coated seeds (77.5%) (Table 1, Figure 1). Thyme oil enhanced seedling root and shoot lengths and dry weights, and decreased root to shoot ratio (Figure 1, Table 1), associated with more a developed second leaf, longer stem, and longer roots (Figure 1) compared to control coated seeds.

### 2.2. Stress Imposition

Since water stress was imposed by restricting the Hoagland’s nutrient water solution, the effect of nutrient restriction is considered to interact with water stress effect in the interpretation of plant response. In optimal conditions, pots received 100% of pot water content with 50% of nutritive supply. After stress imposition, pot water content and nutrient supply reached 60%and 30% respectively, at 10 days post stress (dps), and 30% and 15% respectively, at 17 dps, and since then, stress was prolonged until 25 dps with 30% pot water content and 15% nutritive supply (Table 2). Therefore, we can consider that data at 10, 17, and 25 dps represent the effect of moderate, short-term severe stress and long-term severe stress on plant response, respectively.

### 2.3. The Effects of Seed Coating with Thyme Oil on Root and Shoot Development, Normalized Difference Vegetation Index, Carbon and Nitrogen Content, and the Stable Isotopic Compositions ^13^C and ^15^N, under Well Watered and Water/Nutrient Stress Conditions

Analysis of variance (Table 3) revealed that coating, and irrigation + nutrient supply affected significantly, the shoot and roots’ dry weight, NDVI and root to shoot ratio (*p* < 0.001), while the effect of their interaction was only significant for root to shoot ratio (*p* < 0.01). Effect of coating was not significant (*p* > 0.05) on shoot N and C content but highly significant for shoot δ^13^C (*p* < 0.001), and less significant for shoot δ^15^N (*p* < 0.05). The effect of irrigation + nutrient supply was highly significant for shoot N content and δ^13^C (*p* < 0.001), significant for shoot δ^15^N (*p* < 0.01), and less significant for shoot C content (*p* < 0.05). The effect of interaction coating × (irrigation + nutrient supply) was highly significant for all traits (*p* < 0.001).

**Figure 2:**
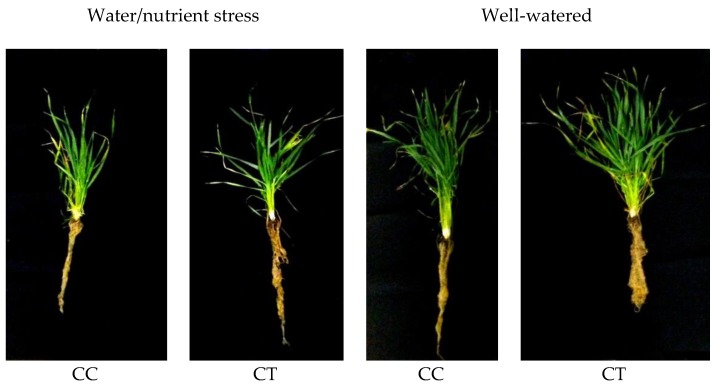
Shoot and root development in wheat plants under well-watered and water/nutrient stress conditions at 25 dps, CC: Coated control; CT: Coated with thyme oil.

In well watered conditions, thyme oil showed a growth promoting effect that included an increase of both shoot and roots areas (Figure 2), and significant increases in roots’ dry weight, root to shoot ratio, and NDVI (Table 3). Thyme oil significantly increased shoot δ^13^C, and decreased shoot N and C content and δ^15^N, compared to the well-watered control. Water/nutrient stress imposition in control plants induced reduction of both root and shoot area (Figure 2) associated with a significant reduction (*p* < 0.005) in root and shoot dry weight with more accentuated reduction in roots (reduction rate (RR) = 71% and 55.3% respectively), root to shoot ratio (RR = 35.7%), and NDVI (RR = 12.5%), (Table 3). In addition, water/nutrient stress significantly increased shoot δ^13^C (IR = 3.15%), and decreased shoot C content (RR = 13.7%), slightly decreased δ^15^N (RR = 14.6%), and increased N content (IR = 5.34%). Under water/nutrient stress, thyme oil reduced the stress-damage effect characterized by smaller reduced shoot and roots areas (Figure 2), a less-reduced root dry weight and NDVI (RR = 51.4% and 9.52% respectively) (Table 3), and a lesser increase in δ^13^C (IR = 1.06%) compared to stressed control plants. Unlike control plants, stress imposition in plants treated with thyme oil increased δ^15^N (IR 29%). Thyme oil induced changes in roots morphology by stimulating their elongation (Figure 2). Values of NDVI and root to shoot ratio were higher in stressed plants treated with thyme oil than well-watered control plants (Table 3).

### 2.4. The Effect of Coating with Thyme Oil on ABA Content under Well-Watered and Water/Nutrient Stress Conditions

**Figure 3:**
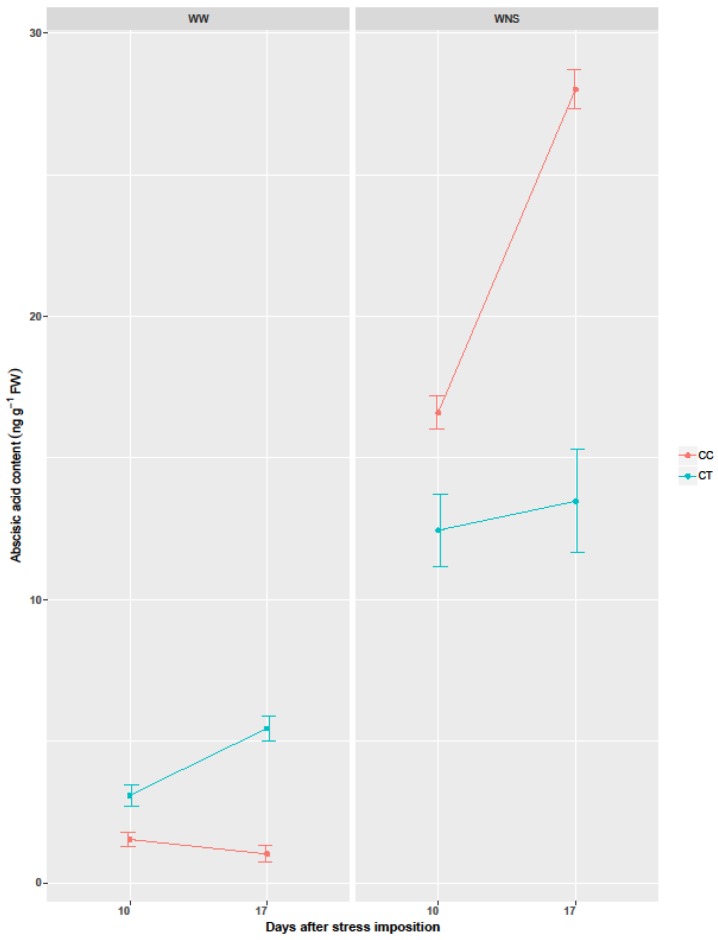
Changes in total endogenous ABA concentrations in leaves of wheat plants either well-watered and water/nutrient stressed, WW: Well watered; WNS: Water/nutrient stress; CC: Coated control; CT: Coated with thyme oil.

ABA content was significantly affected by coating, irrigation, nutrient supply, time of measurements and their interactions (Table 4). In well-watered conditions, endogenous ABA was slightly higher in plants coated with thyme oil while only ABA traces were observed in controls plants (Figure 3). Under water/nutrient stress, endogenous ABA increased with increasing water/nutrient deficit applied to control plants; ABA increased by 15 fold at moderate stress (10 dps, 60% pot capacity (PC)), and by 25 fold at short-term severe stress (17 dps, 30% PC). In plants coated with thyme, water/nutrient stress increased endogenous ABA by 10 folds at moderate stress (10 dps, 60% PC), and was maintained stably (10 folds higher than stressed plants) despite the stress increase at 17 dps (30% PC).

### 2.5. The Effect of Coating with Thyme Oil on Plant Response under Well-Watered and Water/Nutrient Stress Conditions Measured with Dualex

**Figure 4:**
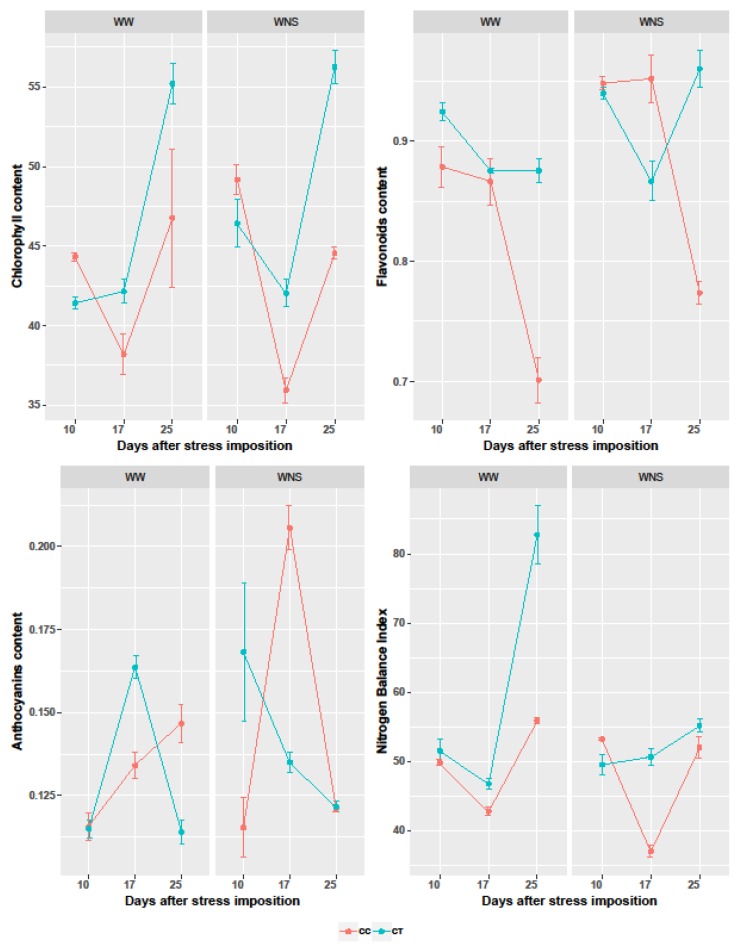
Chlorophyll, flavonoids, anthocyanins, and nitrogen balance index in leaves of wheat plants either well-watered or water/nutrient stressed, WW: Well-watered; WNS: Water/nutrient stress; CC: Coated control; CT: Coated with thyme oil.

Analysis of parameters measured by the Dualex device demonstrated that flavonoids and NBI were significantly affected by coating, irrigation, nutrient supply, and time, whereas chlorophyll was only significantly affected by coating and time, and anthocyanins were only affected by irrigation, nutrient supply, and time (Table 5). In well-watered conditions, thyme oil increased chlorophyll, flavonoids and the nitrogen balance index (NBI) (Figure 4); this effect was clearly visible at the most advanced plant growth stage (25 dps). At moderate stress (10 dps), water- nutrient stress imposition increased chlorophyll and flavonoids’ content in both control and seed coating treatment, with higher increases in flavonoids in the control compared to the seed coated treatment. Contrastingly, water/nutrient stress imposition increased anthocyanins’ content in the control but decreased it in the seed coated treatment. At short-term severe stress (17 dps), decreases in chlorophyll and NBI were observed in control plants accompanied by a peak in anthocyanins’ content, while flavonoids’ content remained unchanged. In plants coated with thyme oil, increasing water and nutrients deficit induced a minor decrease in chlorophyll compared to control plants, and a decrease in both flavonoids and anthocyanins oppositely to control plants, and NBI remained stable. With long-term severe stress at 25 dps, the control plants’ adaptation to water/nutrient stress was observed characterized by restoration towards the original rates of chlorophyll and NBI associated with decreases in flavonoids and anthocyanins. Plants derived from coated seeds with thyme had highly increased chlorophyll, regained the original rates of flavonoids and anthocyanins by increasing flavonoids and decreasing anthocyanins, and had slightly increased NBI.

## 3. Discussion

### 3.1. The Effects of Thyme Oil on Seed Germination, Seedling Development, and Plant Physiology under Well-Watered Conditions

Improving seed germination is a very important criterion to enhance competitiveness of cultivated plants for the efficient use of water and nutrients [7]. Seed priming involves seed treatment with transient stressing agents during the first hydration phase of the priming process within embryos, leading to a stress memory and more efficient adaptation to subsequent episodes of stress [7]. In our previous work we showed that this sample of thyme essential oil contains the monoterpenes carvacrol (81.65%), ρ-cymene (4.26%), γ-terpinene (5.91%), and the sesquiterpene β.-caryophyllene (4.62%) as the major compounds [10]. Indeed, it has been demonstrated that low doses of allelochemicals, including monoterpenes, promote growth and enhance jasmonic acid-mediated dormancy release of target crops by stimulating mild stress responses [12,13]. Interestingly, in previous findings, wheat seeds exposed to exogenous application of carvacrol were found to be able to metabolize it to the less toxic derivates to thymoquinone (TQ) in the endosperm and to TQ and hydrothymoquinone (HTQ) in the embryo at 15 h post treatment, as part of an existing detoxification process by several enzyme systems, as a consequence of the oxidative burst after the intracellular acidification caused by thyme oil [14]. Therefore, the observed stimulation of seed germination and growth promotion by thyme oil is supposed to be the consequence of seed priming, derived from the beneficial effect of the transient acidification of seed endosperms and embryos induced by the monoterpenes present in thyme oil, known to function as trans-membrane carriers of H+ into the cell cytoplasm leading to transient intracellular acidification, increased permeability of membranes, and the generation of ROS [15,16]. The acidification of seed endosperm and embryo has multiple targets inducing multiple effects: (i) Reversing the inhibitory effect of ABA, acidifying the cytosol and stimulating the electrogenic proton pump leading to increased germination, cell enlargement, and elongation of seedling stems and coleoptiles [17,18]; (ii) solubilizing starch, favoring, in this way, the enzymatic hydrolysis of reserve components and the mobilization of macronutrient elements during germination [19]; (iii) weakening of membrane barriers of the endosperm cells, a subsequent fall in turgor pressure and changes in cell wall enzymes’ activity leading to a larger uptake of water which is considered a crucial factor in the interruption of dormancy, and promotion of germination and growth [20].

Coating seeds with thyme oil activated plant signaling at the early vegetative growth by triggering the ABA signaling pathway and increased chlorophyll, flavonoids, nitrogen balance index, and NDVI at a more advanced plant growth stage. This result supports the potential of thyme oil to induce seed priming and increasing seedling establishment at the early stage of germination, and to induce systemic resistance when applied to roots in hydroponics, as reported in our previous study [10]. The induced ABA could explain, at least in part, the growth promoting effect of thyme oil in terms of increasing the area and dry weight of roots and shoots, the root to shoot ratio, and the increase in flavonoids’ content, since elevated abscisic acid (ABA) has been demonstrated to promote root growth and increase the root to shoot ratio [21], indirectly, via preventing excessive accumulation of ethylene [22], and to induce ROS that will immediately activate the antioxidant systems as flavonoids [22]. The increase in shoot δ^13^C along with plant growth promotion supports the reported positive relationship between δ^13^Cand plant growth (measured as a biomass) in wheat [23,24] and other crop species [25], when plants were grown in pots under a given water regime. The effect of thyme on decreasing shoot δ^15^N indicates its potential to enhance uptake and assimilation of nitrogen sources [26]. Both decreases in shoot C and N content coupled with an increase in nitrogen balance index suggest allocation of C and N compounds from shoots to the demanding roots. All these effects indicate that thyme oil has the potential to improve the water and nutrient status of the plant.

### 3.2. The Effect of Water/Nutrient Stress Imposition in Control Plants

The response of stressed control plants differed in function with water/nutrient stress intensity. The moderate stresses inducing anthocyanins, flavonoids, and ABA, highlights, together with an increase in δ^13^C at the end of the experiment, the important role of these mechanisms in the first line of secondary plant defense metabolism, inducing stomatal closure to maintain leaf water content, reducing photoinhibition, osmo-protecting cells, and scavenging ROS to alleviate oxidative damage [27,28]. The unexpected effect of moderate stress on inducing an increase in chlorophyll can be a defensive response to reduce the harmful effects of drought stress [29].When short but severe stress was imposed, chlorophyll content was negatively affected, indicating dissociation of the light-harvesting complexes from photosynthetic reaction centers as an adaptive response to reduce the drought-induced damage to photosynthesis [28]. The observed decrease in chlorophyll associated with the constant flavonoids content resulted in a decrease in nitrogen balance index, defined as the chlorophyll/flavonoids ratio. It was reported that, under optimal conditions, the plant favors its primary metabolism and synthesizes proteins (nitrogen-containing molecules) containing chlorophyll and few flavonoids (carbon-based secondary compounds), resulting in a higher nitrogen balance index, while, in the case of nitrogen deficiency, the plant directs its metabolism towards an increased production of flavonoids resulting in a lower nitrogen balance index [30]. Our results showed that the plants directed their metabolism from chlorophyll synthesis towards anthocyanins instead of flavonoids which is considered to be the result of the combined water/nutrient stress that the plant was subjected to. In fact, both water and nutrient stress are reported to induce leaf senescence, which is promoted by sugar accumulation and senescence-related hormones, such as ABA [20,21,22,23,24,25,26,27,28,29,30,31]. These findings threshold the key role of anthocyanins in both the reduction of photo-oxidative damage, and the delay of the stress-induced leaf senescence in plants growing under water shortage and nutrient deficiency [20].

All these early metabolic changes were most likely involved in a broader response of the plant as stress progressed to more severe levels, which included decrease in plant growth as part of their adaptation mechanisms to endure water/nutrient stress. The morphological adaptation of the cultivar ‘Karim’ included: (i) Reduction of shoot and root development and NDVI as a consequence of a limitation of photo-assimilates [22]; (ii) decline of root to shoot ratio, mainly due to a more adverse effect on root growth than on shoot growth, as a consequence of the interaction of water stress and nutrient deficiency, resulting in partitioning of more dry matter to above–ground biomass [32], in order to increase availability of assimilates for aboveground parts [33,34]. The decrease in shoot C content and increase in water-use efficiency (δ^13^C) is suggested to be related to ABA-mediated stomatal closure implicated by the restriction of diffusive CO_2_ supply to carboxylation sites, reflecting a decrease in internal ^12^C [35]. The slight decrease in shoot δ^15^N (^15^N/^14^N) associated with an almost stable shoot N content at the same time suggests that (i) the stabilized shoot N content is derived from the morphological adaptation of the cultivar encountering the expected effect of nutrient stress on N diminution, through reduction of plant development, and thus plant demand for nitrogen. (ii) The slight decrease in shoot δ^15^N, reflecting a slight increase of ^14^N, could be attributed to the water stress effect, rather than nutrient stress effect, which involves the reduction in N volatilization from leaves through stomata into the atmosphere [36] probably due to ABA-mediated stomatal closure. This possible mechanism is consistent with the increase of δ^13^C related to ABA-mediated stomatal closure.

### 3.3. The Effect of Thyme Oil on Water/Nutrient Stress Resistance

The effect of coating seeds with thyme oil on lowering ABA accumulation under stress and maintaining it, despite the increase of water deficit reveals the potential of thyme oil to minimize the reduction in the amount of available water under water deficit [29]. The decrease in both anthocyanins and flavonoids, accompanied by a slight increase of ABA, suggests that other antioxidative mechanisms are implicated in thyme oil’s mode of action. In fact, our previous findings revealed that thyme oil triggers peroxidases rather than phenolic compounds to scavenge ROS under biotic stress [10]. This hypothesis is strengthened by the evidence that phenolics and peroxidases are negatively related due to the fact that phenolics, especially flavonoids and phenylpropanoids, are oxidized by phenol peroxidases (guaiacol peroxidase) in the H_2_O_2_-scavenging system [37]. Thyme oil minimized chlorophyll damage and encountered anthocyanins accumulation at the beginning of the severe stress indicating protection of the photosynthetic capacity and a hindrance of photo-oxidation damage and prevention of stress-induced leaf senescence [20]; and is believed to be related to the potential of thyme oil to enhance the peroxidase-mediated antioxidative mechanism [10], since ROS scavenging enzymes are reported to have key role in reduction of chlorophyll degradation [22]. Thyme oil also stabilized the leaf nitrogen balance index, despite the 35% decrease in nutrient supply, indicating a balanced chlorophylls/flavonoids ratio reflecting a balanced nitrogen/carbon ratio and demonstrating the healthy status of the plants [30]. When plant adaptation occurred, at 25 dps, chlorophyll increase might reflect either de novo synthesis of chlorophyll or increased density and/or thickness of leaves resulting in higher pigment content per unit area. The observed regain of flavonoids’ content is most likely related to reduction in peroxidases activity [37], indicating alleviation of the oxidative stress. NBI remained stabilized as result from both increases in chlorophyll and flavonoids. The stressed plants treated with thyme oil induced root elongation as part of the seeking-for-water strategy, reduced the decrease of shoot and roots dry matter, and reduced the decrease of root to shoot ratio derived from reduced decrease in roots dry matter, indicating a balanced partitioning of dry matter, and a balanced allocation of assimilates between above-ground and below-ground biomass [32,33]. Moreover, stressed plants treated with thyme oil reduced the decrease of green area, and minimized the side effect reduction in carbon assimilation, most likely via lowering ABA-mediated-stomatal closure. Both increases in shoot N content and δ^15^N enrichment under water/nutrient stress let us suggest an increase in ^15^N rather than a decrease in ^14^N, most likely attributed to preferential uptake of the δ^15^N-enriched nitrogen source NH_4_^+^, rather than a decrease in nitrogen assimilation [38]. This is most likely the strategy adopted by plants treated with thyme oil in order to alleviate the effect of water/nutrient deficiency, since the preferential uptake of NH_4_^+^ is often related to the more efficient utilization of reducing power within the plant. Since NO_3_^−^ is the most oxidized form of N and NH_4_^+^ is the most reduced form, assimilation of NH_4_^+^ into amino acids requires about 10 ATP equivalents per molecule less than assimilation of NO_3_^−^, thus is less costly in terms of energy [39]. These results and hypothesis are consistent with the observed stabilized NBI. With no doubt, further analysis of the effect of thyme oil on NH_4_^+^ and NO_3_^−^ uptake should be undertaken to confirm the latter hypothesis.

## 4. Material and Methods

### 4.1. Plant Material

The experiment was conducted with a widely cultivated Tunisian variety Karim of durum wheat (*Triticum turgidum* L. subsp. *Durum* (Desf) Husn.). The experiment was conducted from October to mid December 2017 in the Experimental Facilities of the Faculty of Biology at the University of Barcelona (Spain).

### 4.2. Seed Coating Treatment

Before sowing into pots, seeds were coated with thyme essential oil (5 ppm).Thyme essential oil was extracted by hydrodistillation from dried aerial parts of *Thymbra capitata* (L.) Cav. (chemotype carvacrol, voucher specimen D 1186-3), and harvested during the flowering stage from the plain of Kef (Tunisia, 36°23′ N, 8°79′E); the obtained essential oil was distributed into 1 mL-amber-glass vials and stored at 4 °C for subsequent use; the chemical composition of the oil was investigated and carvacrol was identified as the major compound according to Ben Jabeur et al. [10]. The concentration of thyme oil was adjusted to 5 ppm before use with adding 0.5% of dimethyl sulfoxide (DMSO) as a solubilizing agent to assure homogenous application of the essential oil. The coating product Agicote Rouge T17 (AEGILOPS Applications, France), specific for cereal seeds, containing propane-1,2-diol (5–10%), polyethylene glycol mono(tristyrylphenyl)ether (5–10%), and 1,2-benzisothiasol3(2H)-one (0.0357%), was used [40]. The coating technique consists of preparing the coating solution mixture containing 40 µL of the coating product and 400 µL of thyme oil at the adjusted dose (water was used as a control). Then, the coating mixture is applied progressively to 10 g of wheat seeds in continuous rotation, until complete adhesion and absorption, to assure homogeneous distribution of the coating mixture among seeds. Prior to evaluation of the effect of coating seeds with thyme oil, the effect of the coating product was evaluated; the positive or negative effects of the coating product on seed germination and seedling growth were not detected and its inertness was assured.

### 4.3. Seed Germination Assay

Coated seeds were sown on Petri dishes with Hoagland medium (1% agar). Plates were placed in a growth chamber at 22 °C ± 2.0 with a 12 h photoperiod. The percentage of seed germination was measured at 7 days post coating. Then, emerging seedlings were transferred to grow in a non-circulating hydroponics system; seedlings were transplanted on plastic boxes filled with Hoagland’s nutrient solutionand oxygenated by oxygen pumps, with a density of 5 seedlings/box;as 3 boxes for each coating treatment. The plants were kept in a growth chamber with 16 h light, a day/night temperature of 22/18 °C, and a relative humidity of 70%. Root and shoot lengths as well as fresh and dry weights were measured at 14 days post coating.

### 4.4. The Pot Experiment

#### 4.4.1. Growth Conditions

Coated seeds were sown in a total of 32 2-L-pots with a density of 3 seeds/pot; (total of 16 pots for each coating treatment). According to Medina et al. [11], the pots contained a mixture of standard substrate:perlite (1:1, *v*/*v*) and were grown, in a controlled environment chamber (Conviron E15; Controlled Environments, Winnipeg, MB, Canada), with a long light period of 16 h, a photosynthetic photon flux density (PPFD) of 350 μmol·m^−2^ s^−1^, a day/night temperature of 23/17 °C, and a relative humidity of 60%. The temperature and relative humidity in the chamber were continuously monitored by Conviron series controllers (CMP3243 Controlled Environments Ltd., Winnipeg, MB, Canada). The pots were rotated three times a week to assure uniform growth conditions in the growth chamber.

#### 4.4.2. Irrigation and Nutritive Supply Conditions

The plants were uniformly irrigated every 2 days with 50% Hoagland’s nutrient solution. At the beginning of the experiment, the maximum soil water content of each pot was evaluated as the difference between pot weight after watering with the excess water drained and the pot dry weight. During the rest of the entire experiment, pots were watered by direct measurements of the pot weight and the water supply was adjusted to the pot water conditions established for each water regime. Half of the pots were cultivated under well-watered conditions (100% pot capacity, PC), while the other half were subjected to an induced water and relative nutrient stress at 25 days post sowing, by progressively restricting Hoagland’s nutrient solution by 10% PC every 2 days. Overall, a total of 8 pots were used, as replications for each coating treatment and irrigation combination. After 10 days post stress (dps) the stressed plants received 60% of water PC and 30% of nutritive supply (moderate stress) and the first plant sampling was made. Then, water was progressively restricted to 30% PC, and nutritive supply to 15% (short-term severe stress), and this irrigation regime was strictly maintained until the end of experiment (long-term severe stress). Average decrease in water content and nutritive supply was measured.

#### 4.4.3. Sampling

**Scheme 1:**
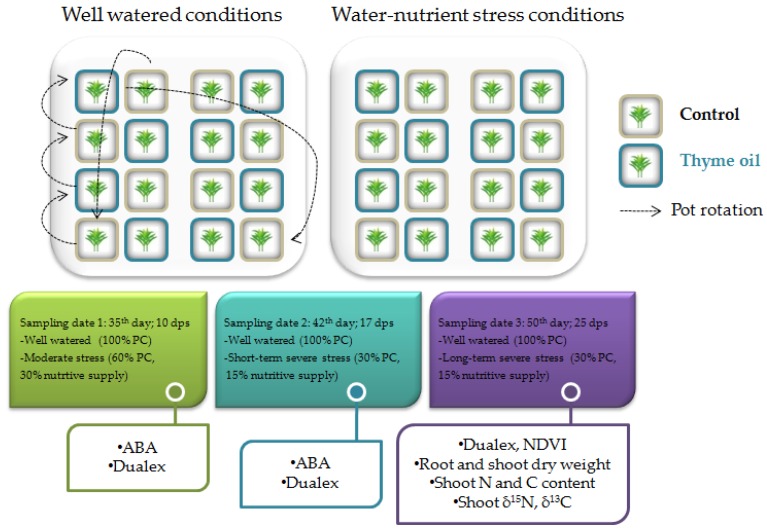
Schematic illustration of the experimental design.

Sampling for abscisic acid (ABA) quantification occurred at 10 and 17 dps, while sampling of total plants for dry weight measurement and isotopic analysis took place at the end of the experiment (25 dps) for both well-watered and water/nutrient stressed plants (see Scheme 1 for a schematic illustration of the experimental design). For dry weight measurements, roots were washed in tap water until all substrate was removed, then root and shoot samples from 25 dps were dried at 70 °C for 48 h.

#### 4.4.4. Leaf Pigments

Chlorophyll (Chl), flavonoids (Flav), anthocyanins (Anth), and the nitrogen balance index (NBI; ratio Chl/Flav) were measured using a portable leaf meter (Dualex Scientific+TM, FORCE-A, France) according to Goulas et al. [30] by selecting 3 measurements at 3 points of the leaf and calculating the mean. In this experiment, measurements were taken at 10, 17, and 25 dps, with 3 replicates per factor combination (coating, irrigation). NDVI values for the individual pots were determined with a portable spectroradiometer (GreenSeeker handheld crop sensor, Trimble, USA) at the end of the experiment at 25 dps. The distance between the sensor and the plots was kept constant at 90 cm from the ground and perpendicular to the plants.

#### 4.4.5. ABA Analysis

Concentrations of ABA were analyzed by liquid chromatography coupled to mass spectrometry in tandem mode (HPLC-MS/MS) according to Thameur et al. [41] with modifications. 50 mg of the last fully expended leaf samples were ground when frozen by liquid nitrogen and the resulting powder was transferred to Eppendorf tubes. To each sample, 500 µL of the extraction solvent isopropanol/methanol/acetic acid (90:9:1, *v*/*v*/*v*) was added, with 20 µL of a solution containing deuterium-labeled abscisic acid (d6-ABA, 0.5 ppm, Saskatoon, Canada) as the internal standard. The extracts obtained were vortexed and centrifuged at 15,000 rpm at 4 °C for 4 min; the supernatants were collected and the pellets were re-extracted with 200 µL of the extraction solvent and centrifuged again. Then, supernatants were pooled, dried with Rotavap for 120 min and reconstituted in 200 µL of methanol/water/acetic acid (90:20:0.01, *v*/*v*/*v*) vortexed, centrifuged (10,000 rpm, 10 min), and filtered through a 0.45mm PTFE filter (Waters, Milford, MA, USA). Finally, 5 mL of each sample was injected into the LC-MS/MS system. A calibration curve was created using serial dilutions of d6-ABA deuterium labeled internal standard.

#### 4.4.6. Shoot Total Carbon and Nitrogen, and Stable Carbon and Nitrogen Isotope Composition

Shoot samples from 25 dps sampling were dried at 70 °C for 48 h and finely ground for further analysis. Approximately 1mg of each sample and reference materials were weighed into tin capsules and measured with an elemental analyzer (Flash1112EA; ThermoFinnigan, Bremen, Germany) coupled with an Isotope Ratio Mass Spectrometer (Delta CIRMS, ThermoFinnigan, Bremen, Germany) operating in continuous flow mode in order to determine the total C and N content and the stable carbon (^13^C/^12^C) and nitrogen (^15^N/^14^N) isotopes’ ratios. The ratios were expressed in δ notation [42], as δ^13^C = [(^13^C/^12^C) sample / (^13^C/^12^C) standard] − 1; where “sample” refers to plant material and “standard” to Pee Dee Belemnite (PDB) calcium carbonate; and as δ^15^N = [(^15^N/^14^N) sample / (^15^N/^14^N) standard] − 1; where “sample” refers to plant material and “standard” to N_2_ in air.

### 4.5. Statistical Analyses

The effects of coating on seed germination, and on root and shoot development of seedlings, were determined through a one-factor analysis of variance (ANOVA) with RStudio 1.1.463 (R Foundation for Statistical Computing, Vienna, Austria). The effect of irrigation, time, coating and their interactions on chlorophyll, anthocyanins, flavonoids, and ABA were determined through a three-factor analysis of variance (ANOVA). The effect of irrigation and coating and their interaction on root and shoot dry weights, root to shoot ratio, NDVI, shoot carbon and nitrogen content, and shoot δ^15^N and δ^13^C isotope composition were determined through a two-factor analysis of variance. When the F-ratio was significant (*p*< 0.05), the least significant difference (LSD) test was used to assess differences between coating/irrigation treatments’ means. Figures for time course variation of chlorophyll, anthocyanins, flavonoids, and ABA were built in the RStudio environment (R Development CoreTeam, 2008).

## 5. Conclusions

To summarize our findings, coating seeds with thyme oil enhanced seed germination, seedling development, and improved the water and nutrient status of the developing plants under well watered conditions. Under stress, coating seeds with thyme oil enhanced the water stress avoidance strategies adopted by the cultivar ‘Karim’ by optimization of water uptake through deep rooting, maintaining better carbon assimilation, and nitrogen uptake. The greater performance of thyme oil was elucidated in its ability to enhance biomass under stress compared to well-watered control plants. Overall, coating seeds with thyme oil is a promising alternative approach to overcome the detrimental effects of drought, tends to be less costly by alternatives, and preserves plant fitness for greater grain filling and yield potential.

## Figures and Tables

**Table 1:** The effects of thyme oil on seed germination and root and shoot dry weights and lengths of wheat plants.

**Traits**	**Germination (%)**	**Shoot Dry Weight (g)**	**Root Dry Weight (g)**
CC	76.16 ^b^ ± 1.25	0.76 ^b^ ± 0.016	0.16 ^b^ ± 0.011
CT	100.00 ^a^ ± 0.0	2.44 ^a^ ± 0.028	0.34 ^a^ ± 0.012
ANOVA Coating	152.4 ***	3289 ***	182.1 ***
**Traits**	**Shoot Length (cm)**	**Root Length (cm)**	**Root/Shoot Ratio**
CC	34.33 ^b^ ± 0.57	19.16 ^b^ ± 0.28	0.21 ^a^ ± 0.01
CT	38.33 ^a^ ± 0.57	41.66 ^a^ ± 1.52	0.14 ^b^ ± 0.003
ANOVA Coating	54.75 ***	323.3 ***	63.38 ***

The F values are shown and the stars indicate statistical significance (*** *p* < 0.001). Values with different superscript letters are significantly different classes according to the least significant difeerence (LSD) test (*p* ≤ 0.05). CC: Coated control; CT: coated with thyme oil.

**Table 2:** Water content and nutritive supply at different time points of the pots submitted to the water and nutrient stress.

	Water Content (%)						Nutritive Supply (%)					
Days after water stress started	6	10	12	14	17	25	6	10	12	14	17	25
Pots containing seeds coated with water (control)	81.0	60.9	41.3	32.5	31.0	30.6	40.5	30.4	20.6	16.2	15.5	15.3
Pots containing seeds coated with thyme oil	80.3	60.3	40.4	30.7	30.1	30.3	40.1	30.1	20.2	15.3	15.05	15.1
Average decrease (%)	19.3	39.4	59.1	68.4	69.4	69.6	9.7	19.7	29.6	34.2	34.7	34.8

**Table 3:** Shoot and root dry weight, NDVI, root to shoot ratio, and shoot nitrogen and carbon abundance and discrimination, under well-watered and water/nutrient stress conditions at 25 days post stress (dps).

**Traits**	**Shoot Dry Weight (mg)**			**Root Dry Weight (mg)**			**NDVI**			**Root to Shoot Ratio**		
**Coating**	**WW**	**WNS**	**RR (%)**	**WW**	**WNS**	**RR (%)**	**WW**	**WNS**	**RR (%)**	**WW**	**WNS**	**RR (%)**
CC	35.89 ^b^ ± 3.83	16.04 ^d^ ± 0.57	55.3%	31.28 ^b^ ± 0.72	9.05 ^d^ ± 0.22	71%	0.16 ^c^ ± 0.00	0.14 ^d^ ± 0.005	12.5%	0.879 ^a^ ± 0.1	0.565 ^b^ ± 0.03	35.7%
CT	44.94 ^a^ ± 0.38	23.40 ^c^ ± 1.42	47.9%	44.32 ^a^ ± 0.68	21.51 ^c^ ± 0.94	51.4%	0.21 ^a^ ± 0.005	0.19 ^b^ ± 0.005	9.52%	0.986 ^a^ ± 0.01	0.921 ^a^ ± 0.06	6.59%
**ANOVA**												
Coating (C)	47.008 ***			1006.094 ***			300 ***			23.182 ***		
Irrigation + nutrient supply (I + N)	298.811 ***			3138.739 ***			33.333 ***			11.870 **		
(T × (I + N))	0.498 ^n.s.^			0.509 ^n.s.^			1.333 ^n.s.^			6.521 *		
**Traits**	**Shoot N content (DM, %)**			**Shoot δ^15^N (‰)**			**Shoot C content (DM, %)**			**Shoot δ^13^C (‰)**		
**Coating**	**WW**	**WNS**	**IR (%)**	**WW**	**WNS**	**IR/RR (%)**	**WW**	**WNS**	**IR/RR (%)**	**WW**	**WNS**	**IR (%)**
CC	3.54 ^b^ ± 0.08	3.74 ^b^ ± 0.11	5.34%	5.06 ^b^ ± 0.26	4.32 ^c^ ± 0.07	RR 14.6%	47.38 ^a^ ± 2.1	40.88 ^b^ ± 0.58	RR 13.7%	30.77 ^c^ ± 0.05	−29.80 ^a^ ± 0.08	3.15%
CT	2.87 ^c^ ± 0.14	4.25 ^a^ ± 0.15	32.2%	4.16 ^c^ ± 0.29	5.86 ^a^ ± 0.07	IR 29%	42.46 ^b^ ± 0.16	45.15 ^a^ ± 1.67	IR 5.9%	30.00 ^b^ ± 0.05	−29.68 ^a^ ± 0.12	1.06%
**ANOVA**												
Coating (C)	1.155 ^n.s.^			7.487 *			0.168 ^n.s.^			86.58 ***		
Irrigation + nutrient supply (I + N)	116.302 ***			17.065 **			5.729 *			187.56 ***		
(T × (I + N))	64.311 ***			108.959 ***			33.363 ***			46.16 ***		

The F values are shown, and the symbols indicate statistical significance (^n.s.^ *p* > 0.1, * *p* < 0.05; ** *p* < 0.01; *** *p* < 0.001). Values with different superscript letters are significantly different classes according to the LSD test (*p* ≤ 0.05). DM: Dry matter; WW: Well watered; WNS: Water/nutrient stress; RR: Reduction rate; IR: Increase rate; CC: Coated control; CT: Coated with thyme oil.

**Table 4:** Result of three-factor ANOVA for abscisic acid (ABA) in coated control plants and plants coated with thyme oil grown under moderate water/nutrient stress and short-term severe water/nutrient stress. The F values are shown, and the symbols indicate statistical significance (*** *p* < 0.001).

Variables	Coating (C)	Irrigation + Nutrient Supply (I + N)	Days Post Stress (dps)	C × (I + N)	C × dps	(I + N) × dps	C × (I + N) × dps
ABA	77.49 ***	1691.9 ***	97.72 ***	291.59 ***	26.98 ***	53.65 ***	84.18 ***

**Table 5:** Results of three-factor ANOVA for chlorophyll, flavonoids, anthocyanins, and nitrogen balance index (NBI) in coated control plants and plants coated with thyme oil grown under moderate, short-term and long-term severe water/nutrient stress.

Traits	Chlorophyll	Flavonoids	Anthocyanins	NBI
Coating (C)	63.291 ***	141.53 ***	1.91 ^n.s.^	207.33 ***
Irrigation + Nutrient supply (I + N)	4.185 ^n.s.^	138.03 ***	27.22 ***	101.57 ***
Time (dps)	155.954 ***	152.42 ***	75.45 ***	356.45 ***
Interaction (C × (I + N))	3.225 ^n.s.^	25.31 ***	0.79 ^n.s.^	37.51 ***
Interaction (C × dps)	53.42 ***	209.13 ***	35.58 ***	77.50 ***
Interaction ((I + N) × dps)	14.261 ***	8.01 **	19.83 ***	97.94 ***
Interaction (C × (I + N) × dps)	0.791 ^n.s.^	11.86 ***	93.97 ***	82.85 ***

The F values are shown, and the symbols indicate statistical significance (^n.s.^ *p* > 0.1, ** *p* < 0.01; *** *p* < 0.001).
